# Extrusion of soybean hulls does not increase digestibility of amino acids or concentrations of digestible and metabolizable energy when fed to growing pigs

**DOI:** 10.1093/tas/txaa169

**Published:** 2020-09-10

**Authors:** Diego A Rodriguez, Su A Lee, María R C de Godoy, Hans H Stein

**Affiliations:** Department of Animal Sciences, University of Illinois, Urbana, IL

**Keywords:** extrusion, feed processing, fiber, pig, soybean hulls

## Abstract

Two experiments were conducted to determine effects of extrusion on energy and nutrient digestibility in soybean hulls. One source of soybean hulls was ground and divided into two batches. One batch was used without further processing, whereas the other batch was extruded. In Exp. 1, four diets were formulated to determine crude protein (CP) and amino acid (AA) digestibility in soybean hulls. A soybean meal-based diet in which soybean meal provided all the CP and AA was formulated. Two diets were formulated to contain 30% nonextruded or extruded soybean hulls and 18% soybean meal. An N-free diet that was used to determine the endogenous losses of CP and AA was also used. Eight growing barrows (initial body weight = 37.0 ± 3.9 kg) had a T-cannula installed in the distal ileum and were allotted to a replicated 4 × 4 Latin square design. Each experimental period lasted 7 d with the initial 5 d being the adaptation period and ileal digesta were collected for 8 h on day 6 and 7. Results indicated that extrusion of soybean hulls did not change the standardized ileal digestibility (SID) of CP and most AA with the exception that the SID of Ile and Leu tended (*P* < 0.10) to be greater in extruded than nonextruded soybean hulls. In Exp. 2, three diets were formulated to determine energy digestibility in soybean hulls. One corn-soybean meal based basal diet, and two diets that contained corn, soybean meal, and 32% extruded or nonextruded soybean hulls were formulated. Twenty-four growing barrows (initial body weight = 59.9 ± 3.4 kg) were allotted to a randomized complete block design. Pigs were housed individually in metabolism crates and feces and urine were collected separately for 4 d after 5 d of adaptation. The apparent total tract digestibility (ATTD) of gross energy (GE) and the digestible energy (DE) and metabolizable energy (ME) were reduced (*P* < 0.05) in diets containing nonextruded or extruded soybean hulls compared with the basal diet. However, the ATTD of GE and values for DE and ME in soybean hulls were not improved by extrusion. Likewise, extrusion did not change the concentration of total dietary fiber in soybean hulls. In conclusion, there were no effects of extrusion of soybean hulls on SID of AA, energy digestibility, or ME concentration in soybean hulls.

## INTRODUCTION

Extrusion may be used to increase the nutritional value of feed ingredients or diets fed to pigs (Liu et al., 2015; Rojas et al., 2016). The use of a combination of heat, pressure, and moisture that is applied to the feed during extrusion may gelatinize starch and improve apparent ileal digestibility (AID) of starch in feed ingredients (Sun et al., 2006; Stein and Bohlke, 2007; Rodriguez et al., 2020) although duration of the extrusion procedure may influence the degree of gelatinization. Extrusion of cereal grains also increased concentrations of metabolizable energy (ME) in corn, and sorghum (Rodriguez et al., 2020) and extrusion may increase the apparent total tract digestibility (ATTD) of gross energy (GE) in high fiber diets (Rojas et al., 2016), indicating that the increase in energy digestibility may be caused not only by increased starch digestibility but also by solubilization of fiber.

Soybean hulls is not an ingredient that is commonly used in swine diets because of the high fiber concentration and the low energy digestibility. However, it was recently reported that soybean hulls may be used in swine diets if diets are pelleted or dietary inclusion is low (Goehring et al., 2019a, 2019b). Likewise, if extrusion increases energy release from fiber, it is possible that soybean hulls can be used in diets for pigs. However, to our knowledge, no data to demonstrate effects of extruding soybean hulls on energy and nutrient utilization by pigs have been reported. Therefore, the objective of these experiments was to test the hypothesis that standardized ileal digestibility (SID) of amino acids (AA), the ATTD of GE, and concentrations of digestible energy (DE) and ME in soybean hulls are increased by extrusion.

## MATERIALS AND METHODS

The Institutional Animal Care and Use Committee at the University of Illinois reviewed and approved the protocol for two experiments. Pigs were the offspring of Line 359 boars and Camborough females (Pig Improvement Company, Hendersonville, TN). One source of soybean hulls was ground and divided into two batches. One batch was used without further processing, whereas the other batch was extruded (Table 1). The extrusion was conducted at the Grain Science and Industry Bioprocessing and Industrial Value Added Products Innovation Center at Kansas State University (Manhattan, KS), using a twin-screw extruder (Wenger TX-52; Wenger Manufacturing Inc., Sabetha, KS). The screw speed was 350 rpm; maximum barrel temperature was 80 °C, and in-barrel moisture was 40%. The extruded product passed through a die that had a diameter of 2.5 mm.

**Table 1.** Nutrient composition of soybean meal, nonextruded soybean hulls, and extruded soybean hulls

		Soybean hulls	
Item, %	Soybean meal	Nonextruded	Extruded
Dry matter	89.0	93.1	91.9
Gross energy, kcal/kg	4,173	3,915	3,892
Crude protein	43.60	9.81	9.67
Acid-hydrolyzed ether extract	2.12	4.84	4.12
Ash	6.27	4.57	4.58
Acid detergent fiber	3.77	44.98	45.12
Neutral detergent fiber	6.89	60.02	58.37
Total dietary fiber	14.2	71.9	71.1
Insoluble dietary fiber	13.5	68.0	63.4
Soluble dietary fiber	0.7	3.9	7.7
Indispensable amino acids			
Arg	3.38	0.47	0.46
His	1.20	0.26	0.26
Ile	2.23	0.42	0.42
Leu	3.55	0.69	0.69
Lys	2.98	0.74	0.70
Met	0.63	0.11	0.11
Phe	2.41	0.40	0.41
Thr	1.81	0.39	0.39
Trp	0.66	0.06	0.10
Val	2.29	0.47	0.47
Dispensable amino acids			
Ala	2.00	0.43	0.43
Asp	5.10	0.97	0.98
Cys	0.63	0.20	0.20
Glu	8.37	1.09	1.11
Gly	1.96	0.84	0.86
Pro	2.44	0.56	0.57
Ser	2.14	0.56	0.58
Tyr	1.74	0.41	0.39
Particle size, μm	750	264	897

### Experiment 1: Ileal Digestibility of Amino Acids

#### Diets, animals, and housing.

Four diets were formulated (Tables 2 and 3) and all diets were in mash form. One diet was a soybean meal-based diet in which soybean meal provided all the crude protein (CP) and AA. Two diets were formulated to contain 30% nonextruded or extruded soybean hulls and 18% soybean meal as the only AA containing ingredients. An N-free diet was also used to determine basal endogenous losses of CP and AA. All diets contained vitamins and minerals to meet or exceed current requirement estimates (NRC, 2012) and all diets also contained 0.40% chromic oxide as an indigestible marker.

**Table 2.** Ingredient composition of experimental diets (as-is basis, Exp. 1)

Item, %	Soybean meal	Nonextruded soybean hulls	Extruded soybean hulls	N-free
Corn starch	53.00	30.35	30.35	68.10
Soybean meal, 43% crude protein	25.00	18.00	18.00	—
Soybean hulls	—	30.00	30.00	—
Sucrose	15.00	15.00	15.00	20.00
Soybean oil	4.00	4.00	4.00	4.00
Solka floc^*a*^	—	—	—	4.00
Magnesium oxide	—	—	—	0.10
Potassium carbonate	—	—	—	0.40
Dicalcium phosphate	1.45	1.60	1.60	2.00
Ground limestone	0.60	0.10	0.10	0.45
Sodium chloride	0.40	0.40	0.40	0.40
Chromic oxide	0.40	0.40	0.40	0.40
Vitamin-mineral premix^*b*^	0.15	0.15	0.15	0.15

^*a*^Fiber Sales and Development Corp., Urbana, OH.

^*b*^The vitamin-micromineral premix provided the following quantities of vitamins and micro minerals per kg of complete diet: vitamin A as retinyl acetate, 11,150 IU; vitamin D_3_ as cholecalciferol, 2,210 IU; vitamin E as selenium yeast, 66 IU; vitamin K as menadione nicotinamide bisulfate, 1.42 mg; thiamin as thiamine mononitrate, 1.10 mg; riboflavin, 6.59 mg; pyridoxine as pyridoxine hydrochloride, 1.00 mg; vitamin B_12_, 0.03 mg; _D-_pantothenic acid as _D-_calcium pantothenate, 23.6 mg; niacin, 44.1 mg; folic acid, 1.59 mg; biotin, 0.44 mg; Cu, 20 mg as copper chloride; Fe, 125 mg as iron sulfate; I, 1.26 mg as ethylenediamine dihydriodide; Mn, 60.2 mg as manganese hydroxychloride; Se, 0.30 mg as sodium selenite and selenium yeast; and Zn, 125.1 mg as zinc hydroxychloride.

**Table 3.** Analyzed composition of experimental diets (Exp. 1)

Item, %	Soybean meal	Nonextruded soybean hulls	Extruded soybean hulls	N-free
Dry matter	90.7	92.1	92.1	91.2
Ash	4.19	4.73	4.60	3.25
Crude protein	11.43	11.02	11.59	0.39
Indispensable amino acids				
Arg	0.80	0.65	0.75	0.01
His	0.29	0.27	0.30	0.01
Ile	0.55	0.50	0.55	0.01
Leu	0.89	0.80	0.89	0.03
Lys	0.72	0.73	0.79	0.02
Met	0.15	0.13	0.14	0.01
Phe	0.60	0.52	0.59	0.02
Thr	0.44	0.41	0.46	0.01
Trp	0.15	0.13	0.16	0.02
Val	0.57	0.52	0.58	0.01
Dispensable amino acids				
Ala	0.50	0.46	0.51	0.01
Asp	1.25	1.12	1.26	0.02
Cys	0.15	0.17	0.18	0.01
Glu	2.07	1.69	1.92	0.03
Gly	0.49	0.59	0.64	0.01
Ser	0.64	0.59	0.67	0.01
Tyr	0.51	0.50	0.56	0.01

Eight growing barrows (initial body weight: 37.0 ± 3.9 kg) were equipped with a T-cannula in the distal ileum (Stein et al., 1998) and allotted to a replicated 4 × 4 Latin square design with four diets and four periods (Kim and Stein, 2009). There were two pigs per diet in each period and a total of eight observations per treatment. Pigs were housed in individual pens (1.2 × 1.5 m) in an environmentally controlled room. Pens had smooth sides and fully slatted T-bar floors. Each pen was equipped with a feeder and a nipple drinker.

#### Feeding and sample collection.

Pigs were fed diets in a daily amount equivalent to three times the maintenance energy requirement (i.e., 197 kcal per kg body weight^0.60^; NRC, 2012), and water was available at all times. Pig weights were recorded at the beginning of each period and at the conclusion of the experiment. The initial 5 d of each period was considered an adaptation period to the diet. On day 6 and 7, ileal digesta were collected for 8 h using standard procedures (Stein et al., 1998) because marker and nutrient concentrations in digesta are stabilized already after 3 d of adaptation (Kim et al., 2020). Collected digesta samples were immediately frozen at −20 °C to prevent bacterial degradation of AA in the digesta. On the completion of one experimental period, animals were deprived of feed overnight and the following morning, a new experimental diet was offered.

#### Chemical analyses, calculations, and statistical analysis.

At the conclusion of the experiment, ileal digesta samples were thawed and mixed within animal and diet, and a subsample was collected for analyses. Ileal digesta samples were lyophilized (Gamma 1–16 LSCplus, IMA Life, Tanowanda, NY) and then finely ground using a coffee grinder.

All samples including soybean meal, soybean hulls, diets, and ileal digesta were analyzed for dry matter (method 930.15; AOAC Int., 2007). Diet and ingredient samples were analyzed for ash (method 942.05; AOAC Int., 2007), and the concentration of CP was calculated as N × 6.25 and N was measured in diets, ingredients, and ileal digesta samples using the combustion procedure (method 990.03; AOAC Int., 2007) on a LECO FP628 (LECO Corp., Saint Joseph, MI). Amino acids in these samples were analyzed on a Hitachi Amino Acid Analyzer, Model No. L8800 (Hitachi High Technologies America, Inc; Pleasanton, CA) using ninhydrin for postcolumn derivatization and norleucine as the internal standard. Prior to analysis, samples were hydrolyzed with 6*N* HCl for 24 h at 110 °C (method 982.30 E(a); AOAC Int., 2007). Methionine and Cys were determined as Met sulfone and cysteic acid after cold performic acid oxidation overnight before hydrolysis (method 982.30 E(b); AOAC Int., 2007). Tryptophan was determined after NaOH hydrolysis for 22 h at 110 °C (method 982.30 E(c); AOAC Int., 2007). Diets and ileal digesta samples were also analyzed for Cr using Inductive Coupled Plasma Atomic Emission Spectrometric method (method 990.08; AOAC Int., 2007) after digestion using nitric acid–perchloric acid (method 968.08D(b); AOAC Int., 2007).

The nonextruded and extruded soybean hulls and the soybean meal were also analyzed for insoluble and soluble dietary fiber using the Ankom Dietary Fiber Analyzer (Ankom Technology, Macedon, NY; method 991.43, AOAC Int., 2007). Acid-hydrolyzed ether extract was analyzed in soybean meal and in the two sources of soybean hulls by acid hydrolysis using 3*N* HCl (AnkomHCl, Ankom Technology) followed by crude fat extraction using petroleum ether (AnkomXT15, Ankom Technology). Particle size of soybean meal and the nonextruded and extruded soybean hulls was determined (ASABE, 2008; de Jong et al., 2016).

Values for the AID and SID of CP and AA were calculated for all diets (Stein et al., 2007). The AID and SID for CP and AA in soybean hulls and extruded soybean hulls were calculated by difference by subtracting the contribution of digestible CP and AA supplied by soybean meal in the diets containing soybean meal and soybean hulls from the total digestible CP and AA in these diets (Adeola, 2001).

Data were analyzed using the PROC MIXED (SAS Inst. Inc., Cary, NC) and homogeneity and normality of the residuals were also confirmed. The model included diet or ingredient as the fixed effect and pig, replicate, and period as random effects. Pig was the experimental unit. Mean values were calculated using the LSMeans statement, and if significant, means were separated using the PDIFF option with Tukey’s adjustment. Results were considered significant at *P* ≤ 0.05 and a trend at *P* ≤ 0.10.

### Experiment 2: Digestibility of GE and Concentration of DE and ME

#### Diets, animals, and housing.

A basal diet containing corn and soybean meal and two diets in which 32% of either extruded or nonextruded soybean hulls were included were formulated (Table 4). Vitamins and minerals were included in all diets to meet or exceed current requirement estimates (NRC, 2012).

**Table 4.** Ingredient composition and analyzed composition of experimental diets (Exp. 2)

Item, %	Basal	Nonextruded soybean hulls	Extruded soybean hulls
Ingredient composition, as-fed basis			
Ground corn	73.30	49.55	49.55
Soybean meal, 43% crude protein	24.40	16.50	16.50
Soybean hulls	—	32.00	32.00
Dicalcium phosphate	0.80	1.00	1.00
Ground limestone	0.85	0.30	0.30
Sodium chloride	0.50	0.50	0.50
Vitamin-mineral premix^*a*^	0.15	0.15	0.15
Analyzed composition			
Dry matter	87.2	87.7	88.4
Ash	4.19	4.43	4.77
Gross energy, kcal/kg	3,845	3,854	3,851
Neutral detergent fiber	7.60	23.64	21.95
Acid detergent fiber	2.44	15.71	14.89

^*a*^The vitamin-micromineral premix provided the following quantities of vitamins and micro minerals per kg of complete diet: vitamin A as retinyl acetate, 11,150 IU; vitamin D_3_ as cholecalciferol, 2,210 IU; vitamin E as selenium yeast, 66 IU; vitamin K as menadione nicotinamide bisulfate, 1.42 mg; thiamin as thiamine mononitrate, 1.10 mg; riboflavin,6.59 mg; pyridoxine as pyridoxine hydrochloride, 1.00 mg; vitamin B_12_, 0.03 mg; _D-_pantothenic acid as _D-_calcium pantothenate, 23.6 mg; niacin, 44.1 mg; folic acid, 1.59 mg; biotin, 0.44 mg; Cu, 20 mg as copper chloride; Fe, 125 mg as iron sulfate; I, 1.26 mg as ethylenediamine dihydriodide; Mn, 60.2 mg as manganese hydroxychloride; Se, 0.30 mg as sodium selenite and selenium yeast; and Zn, 125.1 mg as zinc hydroxychloride.

A total of 24 barrows (initial body weight: 59.9 ± 3.4 kg) were allotted to the three diets in a randomized complete block design with three diets and initial body weight as the blocking factor for a total of eight replicate pigs per diet. Pigs were housed individually in metabolism crates that were equipped with a slatted floor, a self-feeder, and a nipple waterer. A screen and a urine pan were placed under the slatted floor of each crate to allow for the total, but separate, collection of urine and fecal materials.

#### Feeding and sample collection.

Pigs were fed at three times the energy requirement for maintenance. Diets were provided every day in two equal meals at 0800 and 1600 h. Throughout the study, water was available at all times. All diets were provided in a meal form. Diets were fed for 12 d; the initial 5 d were considered the adaptation period to the diet, whereas fecal materials and urine were collected separately from the feed provided from day 6 to day 10 according to the marker to marker approach (Adeola, 2001). Urine was collected in buckets over a preservative of 50 mL of 6*N* HCl. Fecal samples and 20% of the collected urine were stored at −20 °C immediately after collection.

#### Chemical analyses, calculation, and statistical analysis.

At the conclusion of the experiment, urine and fecal samples were thawed and mixed within animal and diet, and a subsample of each urine sample was lyophilized before analysis (Kim et al., 2009). Fecal samples were dried at 50 °C in a forced-air drying oven, and dried samples were ground through a 1-mm screen using a Wiley mill (Model 4; Thomas Scientific, Swedesboro, NJ). Ingredients, diets, ground fecal samples, and lyophilized urine samples were analyzed for GE using bomb calorimetry (Model 6400; Parr Instruments, Moline, IL). Diets were analyzed for DM and ash as explained for Exp. 1. Diets were also analyzed for ADF and NDF using Ankom Technology method 12 and 13, respectively (Ankom 2000 Fiber Analyzer, Ankom Technology).

Following analysis, the ATTD of GE was calculated for each diet and the DE and ME in each diet was calculated as well (Adeola, 2001). The ATTD of GE and concentrations of DE and ME in soybean hulls was calculated by difference (Adeola, 2001). Net energy (NE) in nonextruded and extruded soybean hulls was predicted from ME and analyzed nutrient composition (Noblet et al., 1994). Data were analyzed as explained for Exp. 1.

## RESULTS

Pigs remained healthy during the two experiments and very little feed refusals were observed. Results from Exp. 1 indicated that values for the AID and SID of all AA in the diet containing only soybean meal were greater (*P* < 0.05) than in the two diets containing soybean meal and 30% soybean hulls, with the exception that the SID of Arg was not different among the three diets (Table 5). Extrusion did not change the AID or SID of most AA in diets with the exception that the AID of Leu, Phe, Asp, Ser, and Tyr in the diet with extruded soybean hulls was greater (*P* < 0.05) than in the diet with nonextruded soybean hulls. Likewise, the AID of Leu, Phe, and Tyr was greater (*P* < 0.05), and the AID of Ile Val, Asp, and Ser tended (*P* < 0.10) to be greater in extruded compared with nonextruded soybean hulls (Table 6). The SID of Ile and Leu also tended (*P* < 0.10) to be greater in the extruded soybean hulls compared with the nonextruded soybean hulls.

**Table 5.** AID and SID of CP and AA in diets fed to growing pigs^*a,b*^ (Exp. 1)

	AID					SID				
Item, %	Soybean meal	Nonextruded soybean hulls	Extruded soybean hulls	SEM	*P* value	Soybean meal	Nonextruded soybean hulls	Extruded soybean hulls	SEM	*P* value
CP	68.1^a^	55.5^b^	53.5^b^	3.5	<0.001	86.6^a^	75.0^b^	72.9^b^	3.5	<0.001
Indispensable AA									
Arg	80.7^a^	72.6^b^	75.6^ab^	3.1	0.016	92.5	87.3	88.3	3.0	0.097
His	84.3^a^	70.3^b^	73.2^b^	1.3	<0.001	91.3^a^	78.0^b^	80.0^b^	1.3	<0.001
Ile	83.6^a^	72.3^b^	76.1^b^	1.3	<0.001	90.4^a^	79.9^b^	82.9^b^	1.3	<0.001
Leu	83.4^a^	72.9^c^	76.9^b^	1.2	<0.001	90.3^a^	80.7^b^	83.9^b^	1.2	<0.001
Lys	84.0^a^	70.7^b^	72.7^b^	1.6	<0.001	90.8^a^	77.6^b^	79.1^b^	1.6	<0.001
Met	84.4^a^	76.1^b^	77.9^b^	2.0	0.001	91.3^a^	84.1^b^	85.4^b^	2.0	0.003
Phe	84.2^a^	74.0^c^	77.7^b^	1.1	<0.001	90.9^a^	81.9^b^	84.6^b^	1.1	<0.001
Thr	74.1^a^	63.6^b^	67.3^b^	1.4	0.002	88.2^a^	78.9^b^	81.0^b^	1.4	<0.001
Trp	84.1^a^	76.3^b^	78.9^b^	1.2	<0.001	92.3^a^	86.0^b^	86.8^b^	1.2	0.005
Val	80.5^a^	67.5^b^	71.3^b^	1.3	<0.001	89.7^a^	77.8^b^	80.5^b^	1.3	<0.001
Total	82.2^a^	71.2^b^	74.5^b^	1.3	<0.001	91.1^a^	80.8^b^	83.1^b^	1.3	<0.001
Dispensable AA									
Ala	70.4^a^	55.4^b^	58.4^b^	3.0	<0.001	86.2^a^	72.9^b^	74.1^b^	3.1	0.001
Asp	80.6^a^	69.9^c^	74.0^b^	1.2	<0.001	87.8^a^	78.1^b^	81.3^b^	1.2	<0.001
Cys	68.6^a^	58.6^b^	59.4^b^	1.9	0.001	83.6^a^	72.0^b^	72.0^b^	1.9	<0.001
Glu	85.3^a^	75.8^b^	77.9^b^	1.4	<0.001	90.6^a^	82.3^b^	83.6^b^	1.4	<0.001
Gly	37.2^a^	19.7^b^	20.3^b^	8.7	0.004	80.9^a^	56.7^b^	54.4^b^	8.7	<0.001
Ser	77.9^a^	63.4^c^	67.2^b^	1.4	<0.001	89.9^a^	75.8^b^	78.3^b^	1.4	<0.001
Tyr	80.6^a^	66.7^c^	70.8^b^	1.3	<0.001	89.6^a^	75.8^b^	78.8^b^	1.3	<0.001
Total	60.7^a^	46.8^b^	50.5^b^	5.6	0.001	84.4^a^	73.1^b^	73.9^b^	5.7	0.002
Total AA	70.7^a^	58.0^b^	61.5^b^	3.4	<0.001	87.3^a^	76.6^b^	78.1^b^	3.4	<0.001

^a-c^Within a row, means without a common superscript differ (*P* < 0.05).

^*a*^Each least squares mean is the mean of eight observations, with the exception that data for the diet containing nonextruded soyhulls are the mean are the mean of seven observations.

^*b*^Values for standardized ileal digestibility were calculated by correcting the values for apparent ileal digestibility for basal ileal endogenous losses. Basal ileal endogenous losses were determined (g/kg of DM intake) as CP, 23.30; Arg, 1.04; His, 0.22; Ile, 0.41; Leu, 0.68; Lys, 0.54; Met, 0.11; Phe, 0.44; Thr, 0.68; Trp, 0.14; Val, 0.58; Ala, 0.87; Asp, 1.00; Cys, 0.25; Glu, 1.20; Gly, 2.36; Ser, 0.67; and Tyr, 0.36.

**Table 6.** AID and SID of CP and AA in nonextruded and extruded soybean hulls^*a*^ (Exp. 1)

Item, %	AID				SID			
	Nonextruded soybean hulls	Extruded soybean hulls	SEM	*P* value	Nonextruded soybean hulls	Extruded soybean hulls	SEM	*P* value
CP	47.7	44.5	5.5	0.408	67.8	64.4	5.5	0.388
Indispensable AA								
Arg	66.7	71.8	5.6	0.385	83.5	85.3	5.6	0.762
His	61.4	66.0	2.3	0.137	69.5	72.8	2.3	0.255
Ile	64.7	71.1	2.4	0.055	72.7	78.0	2.4	0.100
Leu	65.8	72.5	2.2	0.036	74.2	79.7	2.2	0.075
Lys	63.1	66.1	2.8	0.380	70.0	72.1	2.8	0.518
Met	69.7	73.4	3.7	0.207	78.6	81.3	3.7	0.338
Phe	66.9	73.2	2.1	0.043	75.6	80.3	2.1	0.107
Thr	56.8	63.0	2.4	0.117	72.9	76.4	2.4	0.331
Trp	70.9	75.4	2.4	0.220	81.6	83.0	2.4	0.682
Val	58.8	65.2	2.3	0.054	69.7	74.4	2.3	0.134
Total	63.9	69.4	2.2	0.097	74.2	78.0	2.2	0.224
Dispensable AA								
Ala	45.7	50.6	4.7	0.391	64.2	66.3	4.7	0.710
Asp	63.0	69.5	2.1	0.064	71.8	76.9	2.1	0.133
Cys	53.8	54.4	2.9	0.874	66.3	65.9	2.9	0.914
Glu	69.2	72.4	2.7	0.374	76.7	78.5	2.7	0.594
Gly	13.1	11.9	11.0	0.893	46.6	41.1	11.0	0.533
Ser	54.6	60.7	2.2	0.055	67.2	71.2	2.2	0.171
Tyr	58.3	64.9	2.1	0.049	67.5	72.3	2.1	0.127
Total	38.9	43.8	8.4	0.445	66.8	67.0	8.4	0.969
Total AA	50.2	55.5	5.0	0.269	70.0	72.0	5.0	0.664

^*a*^Data for the nonextruded soybean hulls are the mean of seven observations and data for the extruded soybean hulls are the mean of eight observations.

Results from Exp. 2 indicated that feed intake and GE intake were not affected by dietary treatments (Table 7). Feces GE output increased (*P* < 0.05) in pigs fed nonextruded or extruded soybean hulls compared with pigs fed the basal diet. However, there was no difference in feces GE output between nonextruded and extruded soybean hulls. The ATTD of GE was reduced (*P* < 0.05) and DE and ME were also reduced (*P* < 0.05) in the diets containing nonextruded or extruded soybean hulls compared with the basal diet. However, the ATTD of GE and concentrations of DE and ME were not different between the two diets containing nonextruded or extruded soybean hulls. Likewise, the ATTD of GE and concentrations of DE, ME, and NE in extruded soybean hulls were not different from values calculated for the nonextruded soybean hulls (Table 8).

**Table 7.** ATTD of GE and DE and ME in experimental diets fed to pigs^*a*^, as-fed basis (Exp. 2)

Item, %	Basal diet	Soybean hulls		SEM	*P* value
		Nonextruded	Extruded		
Feed intake, kg/d	2.07	2.19	2.17	0.07	0.452
GE intake, Mcal/d	7.95	8.44	8.37	0.28	0.427
Dry feces output, kg/d	0.19^b^	0.45^a^	0.48^a^	0.03	<0.001
Feces GE output, kcal/d	914^b^	2,008^a^	2,138^a^	116	<0.001
ATTD of GE, %	88.5^a^	76.2^b^	74.7^b^	0.9	<0.001
DE in diet, kcal/kg	3,402^a^	2,937^b^	2,877^b^	33	<0.001
Urine output, kg/d	8.80	7.84	11.07	1.73	0.385
Urine GE output, kcal/d	268	227	252	31	0.608
ME in diet, kcal/kg	3,273^a^	2,833^b^	2,759^b^	37	<0.001

^a,b^Within a row, means without a common superscript differ (*P* < 0.05).

^*a*^Each least squares mean represents eight observations.

**Table 8.** ATTD of GE and concentrations of DE, ME, and NE in nonextruded and extruded soybean hulls^*a*^ (Exp. 2)

Item	Soybean hulls		SEM	*P* value
	Nonextruded	Extruded		
ATTD of GE, %	49.6	45.1	2.9	0.297
As-fed basis, kcal/kg				
DE	1,941	1,755	114	0.268
ME	1,893	1,662	127	0.219
NE^*b*^	1,020	841	92	0.192
DM basis, kcal/kg DM				
DE	2,086	1,909	123	0.328
ME	2,034	1,808	137	0.262
NE^2^	1,095	915	99	0.221

^*a*^Each least squares mean represents eight observations.

^*b*^Net energy in nonextruded and extruded soybean hulls was predicted from ME and analyzed nutrient composition (Noblet et al., 1994).

## DISCUSSION

Concentrations of nutrients in soybean meal and soybean hulls used in these experiments are in agreement with reported values (Sauvant et al., 2004; NRC, 2012) with the exception that the concentration of acid-hydrolyzed ether extract in soybean hulls was greater compared with previous values (NRC, 2012; Jaworski and Stein, 2017). However, Barbosa et al. (2008) reported that fat concentration in 38 sources of soybean hulls varied between 0.60 and 4.30% and the soybean hulls used in the present experiment had values for acid-hydrolyzed ether extract that where close to the upper values in this range. It is possible that the reason for this variation in fat content among sources of soybean hulls is that some crushing plants add the soap stock to the soybean hulls, which results in increased fat concentration in the hulls.

Pigs that were 37 kg at the start of the experiment were used to measure SID of AA because values for SID of AA do not change for pigs between 20 and 100 kg (Pedersen et al., 2016). It is, therefore, expected that the values obtained in the experiment are representative for growing-finishing pigs greater than 20 kg. Values for SID of AA in SBM that were observed in this experiment were in agreement with published data, but the SID for most AA in soybean hulls was greater than previously reported (Sauvant et al., 2004; Rostagno et al., 2017). It is possible that the greater values for the SID of AA in the soybean hulls used in this experiment were a result of the increased fat concentration in the soybean hulls used in this experiment because increasing concentrations of oil in diets increases AA digestibility (Li and Sauer, 1994; Albin et al., 2001; Cervantes-Pahm and Stein, 2008). The increased SID of AA in diets with greater concentration of fat is likely a result of slower gastric emptying (Gentilcore et al., 2006), which increases the time dietary AA are exposed to proteolytic enzymes, and thus, more dietary AA may be digested and absorbed. The diets containing soybean hulls contained 4% added soybean oil, which may also have contributed to an increased AA digestibility. However, it is also possible that there are differences in SID of AA among sources of soybean hulls.

Extrusion is expected to increase digestibility of protein because of changes in the three-dimensional structure of proteins that are unfolded by heat, which results in increased access to the protein by gastrointestinal enzymes (Freire et al., 1991; Ai, 2013). Indeed, extrusion increased SID of AA in field peas (Stein and Bohlke, 2007) and in mixed diets (Rojas et al., 2016). However, soybean hulls contain much less AA than field peas and mixed diets, which may be the reason no increase in digestibility of AA was observed after extrusion in this experiment.

The values for DE and ME in the basal diet were in agreement with calculated values for DE and ME in a corn-soybean meal diet (NRC, 2012). The ATTD of GE obtained in the two diets containing corn, soybean meal, and soybean hulls was also in agreement with reported data (Stewart et al., 2013; Jaworski and Stein, 2017). Likewise, the ATTD of GE calculated for the two sources of soybean hulls used in this experiment (49.6 and 45.1%) was in very good agreement with Kornegay (1978) and Jaworski and Stein (2017), who reported ATTD values for GE in soybean hulls of 47.5 and 46.9%, respectively. Even though the soybean hulls that were used in this experiment contained more fat, the DE and ME values for soybean hulls that were generated in this experiment were also within the range of reported values (Sauvant et al., 2004; Barbosa et al., 2008; NRC, 2012). The NE (1,020 and 841 kcal/kg for nonextruded and extruded soybean hulls) was also in agreement with the NE for soybean hulls fed to finishing pigs (i.e., 852 kcal/kg) that was reported by Stewart et al. (2013), and close to values from Sauvant et al. (2004) and NRC (2012) who reported NE in soybean hulls of 1,004 and 989 kcal/kg, respectively.

Digestibility of GE and DE and ME in feed ingredients may be increased by extrusion because extrusion gelatinizes starch, which results in an improvement in starch digestibility (Ai, 2013; Rodriguez et al., 2020). However, the observation that there was no effect of extrusion on energy digestibility in soybean hulls may be because soybean hulls do not contain starch (Sauvant et al., 2004; NRC, 2012). The decreased ATTD of GE that was observed as soybean hulls were included in the diets is in agreement with previous data (Stewart et al., 2013) and likely is a result of the increase in fiber concentration in diets containing soybean hulls, which reduced energy digestibility.

Including up to 20% soybean hulls in diets fed to pigs has negative effects on growth of pigs (Goehring et al., 2019a), which may be a result of the low energy digestibility although less than 5% soybean hulls at the expense of corn in nursery diets did not affect growth performance of pigs (Goehring et al., 2019b). However, the negative effects of soybean hulls in swine diets may be ameliorated by pelleting the diets (Goehring et al., 2019a). Extrusion did not change the concentration of total dietary fiber in soybean hulls, but there was a small increase in soluble dietary fiber and a corresponding reduction in insoluble dietary fiber in the extruded soybean hulls compared with the nonextruded soybean hulls. Although soluble dietary fiber has a greater ATTD than insoluble dietary fiber (Urriola et al., 2010; Navarro et al., 2018), the increase in soluble dietary fiber that was observed as a result of extrusion of the soybean hulls likely was too small to result in a measurable effect on the ATTD of GE. This is likely the reason for the lack of an increase in DE, ME, and NE in the extruded soybean hulls compared with the nonextruded soybean hulls. However, this response is in contrast with data indicating that extrusion of a diet containing corn, soybean meal, distillers dried grains with solubles, and soybean hulls resulted in a greater increase in ME than extrusion of a corn-soybean meal diet (Rojas et al., 2016). However, the extruder used in the study by Rojas et al. (2016) was a single screw Bühler extruder that was operated at 115 °C, whereas a double screw Wenger extruder operated at 80 °C was used in the present experiment. It is also possible that the different results is a consequence of the fact that the entire diet including corn, soybean meal, and distillers dried grains with solubles was extruded in the study by Rojas et al. (2016), whereas in the current experiment, only the soybean hulls were extruded.

## CONCLUSION

There were only minor effects of extrusion on the AID and SID of AA in soybean hulls. Likewise, the ATTD of GE, and the DE, ME, and NE in soybean hulls did not increase by extrusion. However, results from this experiment indicate that the SID of AA in soybean hulls containing 4% to 5% acid-hydrolyzed ether extract may be greater than previously reported for soybean hulls containing less acid hydrolyzed ether extract.
